# Use of lanthanides to alleviate the effects of metal ion-deficiency in *Desmodesmus quadricauda* (Sphaeropleales, Chlorophyta)

**DOI:** 10.3389/fmicb.2015.00002

**Published:** 2015-01-28

**Authors:** Franz Goecke, Celia G. Jerez, Vilém Zachleder, Félix L. Figueroa, Kateřina Bišová, Tomáš Řezanka, Milada Vítová

**Affiliations:** ^1^Laboratory of Cell Cycles of Algae, Centre Algatech, Institute of Microbiology Academy of Sciences of the Czech RepublicTřeboň, Czech Republic; ^2^Department of Ecology, Faculty of Sciences, University of MálagaMálaga, Spain; ^3^Department of Microbiology, Institute of Microbiology Academy of Sciences of the Czech RepublicPrague, Czech Republic

**Keywords:** algae, toxicity, calcium, manganese, metal requirements, rare earth elements

## Abstract

Lanthanides are biologically non-essential elements with wide applications in technology and industry. Their concentration as environmental contaminants is, therefore, increasing. Although non-essential, lanthanides have been proposed (and even used) to produce beneficial effects in plants, even though their mechanisms of action are unclear. Recently, it was suggested that they may replace essential elements. We tested the effect of low concentrations of lanthanides on the common freshwater microalga *Desmodesmus quadricauda*, grown under conditions of metal ion-deficiency (lower calcium or manganese concentrations). Our goal was to test if lanthanides can replace essential metals in their functions. Physiological stress was recorded by studying growth and photosynthetic activity using a pulse amplitude modulation (PAM) fluorimeter. We found that nutrient stress reduced parameters of growth and photosynthesis, such as maximal quantum yield, relative electron transport rate, photon capturing efficiency and light saturation irradiance. After adding low concentrations of five lanthanides, we confirmed that they can produce a stimulatory effect on microalgae, depending on the nutrient (metal) deprivation. In the case of a calcium deficit, the addition of lanthanides partly alleviated the adverse effects, probably by a partial substitution of the element. In contrast, with manganese deprivation (and at even lower concentrations), lanthanides enhanced the deleterious effect on cellular growth and photosynthetic competence. These results show that lanthanides can replace essential elements, but their effects on microalgae depend on stress and the nutritional state of the microalgae, raising the possibility of environmental impacts at even low concentrations.

## Introduction

Under normal conditions, the concentrations of essential metals inside any living cell are maintained within specific ranges. If the concentration of any biogenic metal is below a lower threshold level, organisms suffer from this metal ion-deficiency, but, on the other hand, an excessive amount of those metals usually turns toxic (Pakrasi et al., 2001). If the metals are not essential, the response is not so obvious. Numerous papers have reported that under particular conditions, elements that are not essential for (higher) plants can stimulate their growth and development (Kastori et al., 2010). Among non-essential heavy metals, lanthanides (Ln), also known as “rare earth elements” (REEs), have been demonstrated to exhibit diverse physiological effects on plants and animals (Wang et al., 2003), and therefore, in countries like China, for the last 30 years, they have been used as fertilizers (Hu et al., 2004). Despite their names, the content of REEs in the Earth's crust is close to 0.015% (Kastori et al., 2010) and their total concentration matches that of copper, lead or zinc (Tyler, 2004; Brown et al., 1990; Hu et al., 2004 and in text references). Rare earth elements are, however, dispersed and not often found concentrated as minerals that are easily exploitable ore deposits. It was the very scarcity of these minerals ores (previously called “earths”) and the difficulties to isolate them that led to the term “rare earth.” Only some countries (India, South Africa) have sufficient deposits to produce rare earth concentrates, however, more than 95% of REE deposits are located in China. Paradoxically, due to their increasing agricultural and industrial uses, the concentration of these elements as environmental pollutants has risen (Loell et al., 2011). As other REEs, lanthanides were generally considered to exert low toxicity (Brown et al., 1990; Wang et al., 2003). Only recently, studies have focused on the ecological effects of Ln and their potential to affect life (Li et al., 2010, and references therein). Those experiments have shown that effects of Ln on plant growth are diverse and dose–response relationships are complicated (Li et al., 2011). Algae, as primary producers and the basis of many food webs, are important and sensitive organisms with opportunities for exposure to Ln, although the effects of Ln on algae are poorly understood. Recently, stimulatory effects of low concentrations of Ln on microalgae were demonstrated at low concentrations, and toxic effects were seen at higher concentrations (Hu et al., 2001; Jin et al., 2009; Tai et al., 2010). However, the mechanisms of action of microalgal growth-promoting factors are still unknown and it is not clear whether the positive effect of Ln is due to their alleviation of symptoms of metal deficiency, as suggested previously for plants (see Wei and Zhou, 2000; Tyler, 2004; Gong et al., 2011), or if the elements participate in other physiological reactions.

There are a few experiments that present evidence that Ln addition, under metal-deficient conditions, could alleviate the symptoms of deficiency by partly substituting for essential elements (e.g., Ca^2+^, Mg^2+^, or Mn^2+^). Elements such as divalent cations like Ca^2+^ and Mn^2+^, among others, have essential biochemical and structural functions in plants and algae. They are involved in countless physiological processes such as signaling pathways, the activity of key enzymes and for the synthesis of several molecules necessary for growth and development (Adam and Issa, 2000; Galon et al., 2010). Therefore, their concentration in the environment can be important limiting factor for the survival of different species, not only in soil but also in aquatic environments (Brand et al., 1983; Jones and Ricciardi, 2005).

Ln^3+^ can interact with a great number of biological macromolecules to form stable complexes, and its combinatorial ability is much higher than that of other divalent cations such as Ca^2+^; it could therefore, to a certain extent, substitute for Ca^2+^ in some biological functions (Squier et al., 1990). Unfortunately, almost all of these experiments were carried out on higher plants (and other macroorganisms).

The first element of the Ln group in the periodic table is lanthanum (La^3+^). Because the ionic radius is similar to, and its valence higher than Ca^2+^, it has been referred to as “*super-calcium*” (Brown et al., 1990). La-ions can act as Ca^2+^ antagonists by displacing Ca^2+^ and binding with stronger affinity to multiple receptors. But as well as blocking Ca^2+^-channels, La^3+^ has been reported to mimic, for example, the Ca^2+^ effect on ion transport in plants (Wei and Zhou, 2000). Therefore, by using La^3+^, it may be possible to alleviate calcium (or other metal) deficiencies and stimulate the growth of plants that are exhibiting symptoms of ion-deficiency. The same applies to the other chemically similar lanthanides.

A few studies have presented evidence of Ln-alleviation of metal-deficient conditions in plants such as maize, oilseed rape, spinach and sunflower (e.g., De la Fuente, 1984; Ni et al., 2004; Huang et al., 2008a; Gong et al., 2011; Zhao et al., 2012). In these publications, different metabolic processes were involved, such as photosynthesis (light and dark reactions), respiration, nitrogen metabolism, and oxidative stress (Table 1). In one unique study, based on the green macroalga *Chara corallina*, lower concentrations of La^3+^ were shown to partially compensate for Ca^2+^-deficiency, thus permitting cytoplasmic streaming (Li et al., 2011). To our knowledge, there are no microalgal studies dealing with metal alleviation by Ln. If a plant or alga is able to replace essential metal ions with other ions for important metabolic processes, even if only partly, it presumably represents an ecological advantage when growing under deficit conditions.

**Table 1:** **Experiments on the effects of plants under metal deficiency and by exposure to lanthanides**.

**Plant species**	**Metal-def**.	**Ln**	**Physiological effects**	**References**
Sunflower (*Helianthus annus*)	Ca^2+^	La^3+^	> Auxin transport	De la Fuente, 1984
Rape (*Brassica napus*)	Ca^2+^	Nd^3+^	+ Roots and seedlings	Wei and Zhou, 2000
			< Membrane lipid peroxidation	Ni et al., 2004
			> Absorption of nutrients	
			> Oxidizing capacity	
Spinach (*Spinacia oleracea*)	Mg^2+^	Ce^3+^	> Chlorophyll	Yin et al., 2009
			> Synthesis proteins	Ze et al., 2009a,b
			> Key enzymes CO_2_ assimilation	
			> Expression of *rbc*L, *rbc*S, *rca*	
			> Oxidative stress resistance	
			> Growth	
	Ca^2+^	Ce^3+^	< Electron transport	Huang et al., 2008a
			> Membrane permeability	Huang et al., 2008b
			> O_2_ evolution rate	Liu et al., 2008, 2009
			> Reactive oxygen species	
			> Phosphorylation	
			> Mg^2+^ATPase, Ca^2+^ATPase	
			> Rubisco	
Maize (*Zea mays*)	Mg^2+^	Ce^3+^	< Chlorophyll synthesis	Zhou et al., 2011
			> N_2_ and carbon assimilation	Zhao et al., 2012
			> PSII activities	
			> Growth	
	Mn^2+^	Ce^3+^	> Chlorophyll biosynthesis	Gong et al., 2011
			> O_2_ evolution rate	Qu et al., 2012
			> Key enzymes CO_2_ assimilation	
			< Photochemical reactions	

Pulse amplitude modulated (PAM) fluorometry is a valuable tool to detect physiological stress and to measure photosynthetic efficiency (Baker, 2008; White et al., 2011; Giovanardi et al., 2014). Photosystem II (PSII) is very sensitive to changes in the environment, and under unfavorable or stressful environmental conditions, such as strong light, high concentrations of salt, or low or high temperatures, the activity of PSII declines rapidly (measured as changes in the maximal quantum yield of PSII photochemistry, *F*_*v*_/*F*_*m*_), so this parameter is often one of the earliest and most sensitive indicators of physiological stress (Figueroa et al., 2003; Hou and Hou, 2013). Phytoplankton, growing under non-stressed conditions, normally presents relatively constant values of *F*_*v*_/*F*_*m*_ in ranges from 0.6 to 0.7; a decrease in these values is interpreted as a stress condition (White et al., 2011; Samorí et al., 2013; Giovanardi et al., 2014).

The aim of this study was to explore the potential effects of Ln on the model green microalga *Desmodesmus quadricauda*. Specifically, we aimed to investigate whether there were beneficial effects of exposure of algae to low concentrations of the metals, and to determine whether Ln can replace essential metals (Mn and Ca) in their functions. We present here the first results of studies on metal-deficiency alleviation by Ln using freshwater microalgae, but we also report on a potential detrimental effect on these primary producers, at Ln concentrations that are usually considered as innocuous.

## Materials and methods

### Microalgal strain and culture conditions

The experimental organism *D. quadricauda* (Turpin) Brébisson (previously known as *Scenedesmus quadricauda*), strain Greifswald/15, was obtained from the Culture Collection of Autotrophic Organisms, Institute of Botany (CCALA, Czech Acad. Sci., Třeboň). The microalga was cultivated under controlled experimental conditions of temperature (30°C) and continuous light in liquid mineral medium (*ŠS-medium*, Zachleder and Šetlik, 1982, Table 2), in laboratory scale tubular photobioreactors, as described previously in Vítová et al. (2011), (hereafter referred as “standard medium”). Cultures were aerated with air containing 2% carbon dioxide (v/v). The photobioreactor was illuminated from one side by fluorescent lamps (Osram DULUX L, 55W/840, Italy) at a surface incident irradiance of 500 μmol m^−2^ s^−1^. The starting pH of the suspension was 7.03 varying during 3 days between 6.9 and 7.2; final value of pH at the end of the third day was 6.9.

**Table 2:** **Composition of the *ŠS-medium* (Zachleder and Šetlik, 1982)**.

**Compound**	**Weight (g L^−1^)**	**Molar units (μmol L^−1^)**	**Element**	**Molar units (μmol L^−1^)**
KNO_3_	2.021	19990.11	N	19999.11
K_2_HPO_4_	0.14	803.67	P	3301.8
KH_2_PO_4_	0.34	2498.16	K	24095.6
MgSO_4_·7H_2_O	0.99	4008.11	Mg	4008.1
			S	4030.0
CaCl_2_·2H_2_O	0.011	74.83	Ca	74.83
			Cl	149.16
FeNaEDTA	0.018	49.05	Fe	49.05
			Na	49.05
H_3_BO_3_	0.003	48.54	B	48.54
ZnSO_4_·7H_2_O	0.00143	4.97	Zn	4.97
MnSO_4_·4H_2_O	0.0012	5.38	Mn	5.38
CuSO_4_·5H_2_O	0.00124	5.35	Cu	5.35
CoSO_4_·7H_2_O	0.0014	6.17	Co	6.17
(NH_4_)_6_Mo_7_O_24_·4H_2_O	0.00184	1.49	Mo	10.4

All compounds are given in grams and molar units. The total content of each element in medium (with the exception of oxygen, hydrogen, and carbon) is given in molar units.

The compositions of metal-deficient nutrient solutions (deficient controls) were similar to the standard medium (Table 2) with the following equivalent modifications: (a) Mn^2+^-deficient medium was prepared by replacement of MnSO_4_ with 8.44 μmol L^−1^ (1.20 mg L^−1^) Na_2_SO_4_; (b) Ca^2+^-deficient medium was prepared by replacement of CaCl_2_ with 256.6 μmol L^−1^ (15 mg L^−1^) NaCl (Table 2).

To prepare cultures limited for Mn or Ca, the initial inoculum (from the agar plate) contained approximately 0.5 μg of algal biomass resuspended in 100 mL Mn- or Ca-free medium to a known final cell concentration (absorbance = 0.1). The element-limited cultures were cultivated for 2 weeks under the same conditions as control cultures. Finally, before the start of the experiment, the medium was again removed and replaced with Ca- or Mn-free medium. Changes in the concentrations of Ca and Mn during preparation of starved cultures were measured by ICP-MS, see Section Determination of Element Content (ICP-MS).

We tested different Ln (lanthanum, cerium, neodymium, gadolinium, and europium) that belong to the group of light rare earth elements (LREE). In the case of Ca^2+^-deprivation experiments, we used a reduced concentration of salt (2.5 mg L^−1^), which was equivalent to 5.65 μmol L^−1^ of Eu, 5.72 μmol L^−1^ of La, 5.76 μmol L^−1^ of Nd, 5.74 μmol L^−1^ of Ce, and 5.61 μmol L^−1^ of Gd. For the Mn^2+^-deprivation experiments, we used a manganese-equivalent concentration of salt (0.5 mg L^−1^), which was equivalent to 1.11 μmol L^−1^ of Eu, 1.13 μmol L^−1^ of La, 1.09 μmol L^−1^ of Nd, 1.14 μmol L^−1^ of Ce, and 1.07 μmol L^−1^ of Gd. In the second case, the concentration was equivalent to the amount of Mn added to the standard medium (Table 2). This was not the case for the Ca^2+^ concentration due to a potential risk of Ln toxicity (determined from a prior test, see below Concentration Range Finding Experiment). For that purpose we used analytical grade (99%, Sigma-Aldrich) chloride compounds (LaCl_3_, CeCl_3_, EuCl_3_, NdCl_3_, and GdCl_3_).

### Concentration range finding experiment

We performed several prior tests (standard medium + Ln) to observe the reaction of *D. quadricauda* to different amounts of Ln, in order to determine the range of concentrations to use in our photobioreactor experiments. Therefore, the experiments were carried out in polystyrene 96-well microplates (NuncMicroWell 96-Well Microplates, Thermo Fisher Scientific Inc., Germany) with flat bottom wells of 300 μL. Each well-received 200 μL of test solution, a nutrient spike (10 μL) and an algal inoculum (10 μL), with a final volume per well of 220 μL (Blaise and Vasseur, 2005). Peripheral wells were filled with distilled water to reduce evaporation. Eight different metal concentrations were tested, and five replicates per test solution were performed, from the highest to the lowest concentration. The negative control was the respective medium + algae + water (no metal), and we incorporated a blank well (medium + elements + water, no algae) for each metal concentration tested. The microplates were incubated under controlled experimental conditions of temperature (30°C) and continuous light (100 μmol m^−2^ s^−1^) in an incubation cabinet (CLF Plant Climatics CU-22L, Germany). In this case, microalgal growth was measured by absorbance at 750 nm at 0, 24, 48, and 72 h, using an automated microplate reader (Infinite^®^ F200, Tecan, US) controlled by Tecani-control software (Tecan Group Ltd.). Microplates were shaken for 10 s in the microplate reader before measuring the absorbance. A calibration curve to establish the relationship between absorbance on the microplate and cell density of *D. quadricauda* was previously performed for various stages of the culture.

### Determination of element content (ICP-MS)

For the determination of Ca, Mn, and Nd in the nutrient medium, the ICP-MS analytical method was used. ICP-MS measurements were performed using an Elan DRC-e (Perkin Elmer, Concord, Canada) equipped with a concentric PTFE nebuliser, a cyclonic spray chamber, a high-efficiency quartz torch and a dynamic reaction cell (DRC) for the elimination of spectral interference. The IS solution for the total metals concentration contained Ca and Mn (2.5 mg/L) in dilute (1:100) HNO_3_ (Suprapur, Merck), and 0.25 mg/L of Nd. Distilled and demineralised water (Millipore, Bedford, MA, USA) was used to prepare all solutions. Samples were passed through a 0.45 μm nylon syringe filter and diluted 1:10 using water.

### Growth kinetics for the photobioreactor

Growth was observed by counting cells using a Bürker chamber under a light microscope. Observations under transmitted light were carried out using a BX51 microscope (Olympus, Japan). Values were expressed in number cells mL^−1^. Dry weight was determined gravimetrically. Aliquots (2 mL) of samples were harvested from the photobioreactors in pre-weighed tubes by centrifugation. The supernatant was discarded and pellets were dried at 105°C until they reached a constant weight (Giovanardi et al., 2014).

For the current experiment, samples were taken every 24 h for 3 days; although PAM samples were taken twice a day (every 24 and 30 h) (see Section Photosynthesis as Chlorophyll Fluorescence Measurements). Every treatment was independently replicated three times. To check that the microalgae were not seriously damaged by the deficit conditions, we carried out an experiment where we separately replaced the deficient medium with the original standard nutrient medium (hereafter as “recovery condition” or Rec).

**Figure 1:**
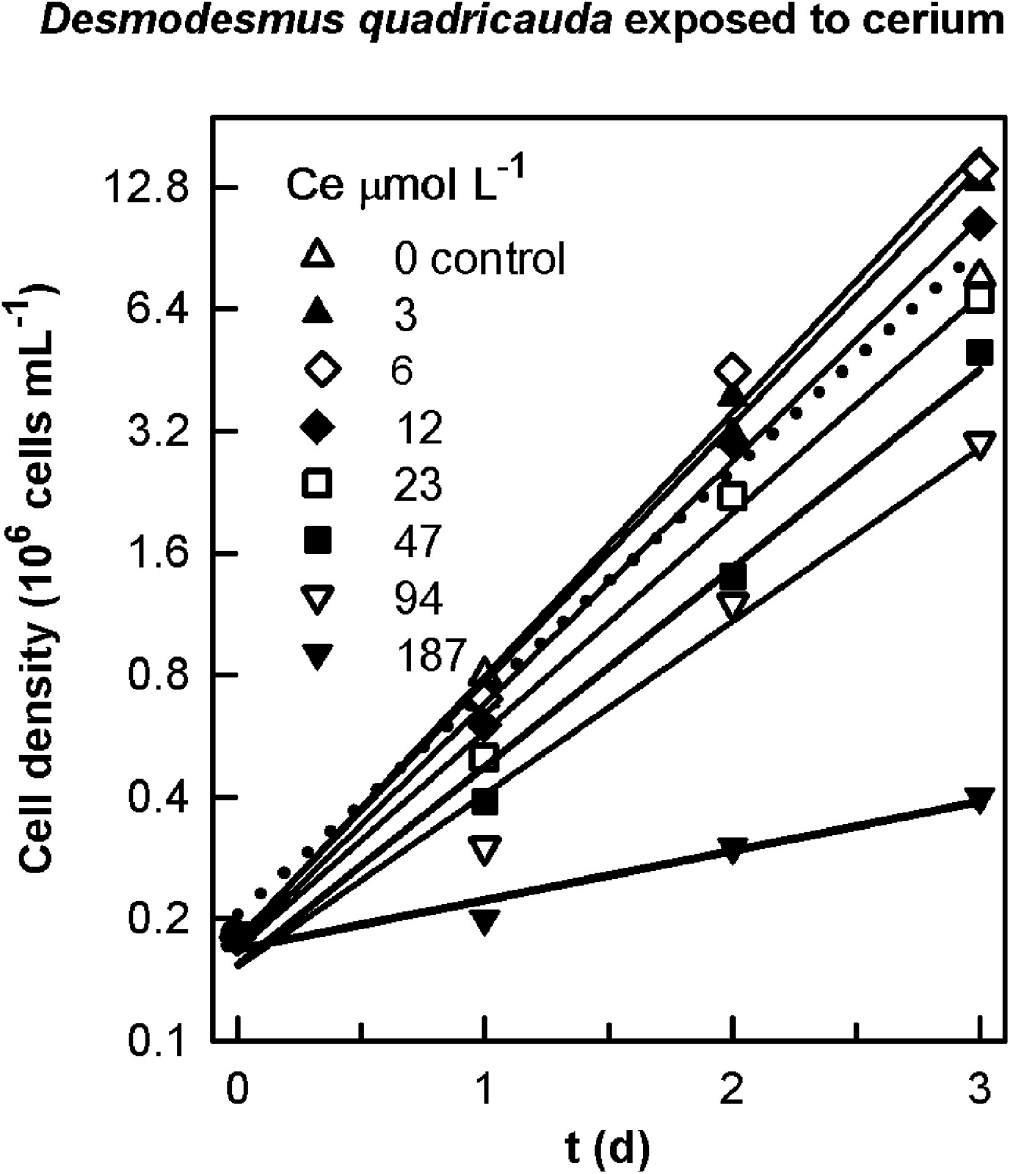
**Changes in cell number in cultures of the alga *Desmodesmus quadricauda* grown in the presence of different concentration of cerium**. The suspensions of cultures were grown in a 96-well microplate for 3 days.

Specific growth rates in the preliminary experiment (Table 3) were determined from the slopes of linear regressions of the natural log of cell concentration vs. time for the data plotted in Figure 1.

**Table 3:** **The effect of cerium concentration on specific growth rates of cultures of *Desmodesmus quadricauda***.

**Cerium concentration (μmol L^−1^)**	**Specific growth rate (μ)(cells 10^6^ L^−1^ day^−1^)**
0	1.27
3	1.44
6	1.47
12	1.36
23	1.19
47	1.12
94	1.03
187	0.28

### Photosynthesis as chlorophyll fluorescence measurements

*In vivo* chlorophyll *a* fluorescence was determined using a Junior-PAM fluorimeter (Walz GmbH, Effeltrich, Germany), provided with blue light emitting diodes for measuring excitation and actinic light. Rapid light curves (RLCs) were carried out twice per day by sampling 5 mL of culture and transferring this to light-protected chambers for dark adaptation (15 min) in order to measure F_*o*_ (basal fluorescence in dark adapted samples). After that, a saturating flash (600 ms ~ 9000 μmol m^−2^ s^−1^) was applied in order to obtain maximal fluorescence (F_*m*_). Maximal quantum yield (*F*_*v*_/*F*_*m*_) was calculated according to Schreiber et al. (1986) and the effective quantum yield (ΔF/F'_*m*_) was calculated as ΔF/F'_m_ = (F'_m_ – F_t_)/F'_m_ (Schreiber et al., 1995b), where F'_m_ represents the maximal fluorescence and F_t_ the current steady-state fluorescence in light adapted algae. Samples were exposed for 20 s to twelve increasing E_PAR_ levels between 0 and 1500 μmol photons m^−2^ s^−1^ to conduct RLCs according to Schreiber et al. (1995a). Relative electron transport rates (rETR, μmol electrons m^−2^ s^−1^) were computed by multiplication of ΔF/F_m_' and the incident irradiance (E, μmol photons m^−2^ s^−1^) as given by the Junior-PAM. rETR values were fitted according to Eilers and Peeters (1988) using a least squares error calculation and the Solver function of Excel (Microsoft, Redmond, U.S.A.) in order to obtain photosynthetic parameters i.e., photon-capturing efficiency of PSII in the light limited range (α), maximum rETR (rETR_max_), and the light saturation irradiance (E_k_).

### Statistical analysis

All experiments were repeated at least twice. Two different Two-Way ANOVA analyses were performed: (1) to determine significant differences (*p* < 0.05) among Ca^2+^ and Mn^2+^ treatments and (2) to determine significant differences (*p* < 0.05) between treatments and controls. In the case of significant effects, the Student–Newman–Keuls *post-hoc* test was applied (Underwood, 1997). Three replicates (*n* = 3) of each nutrient treatment and controls were used for each sampling time. The software Statistica for Windows (version 7.0, Statsoft, Inc., 1984–2004) was used for analyses. Data were presented as means ± SD.

## Results

### Concentration range-finding experiment

In prior rapid tests to establish the correct concentration range, we confirmed that non-essential Ln produce biological effects on algae and have demonstrated stimulatory and toxic effects on growth at lower (<25 μmol L^−1^) and higher concentrations (>25 μmol L^−1^), respectively (Figure 1).

In one graphic example of exposure to Ln, we showed that concentrations of cerium of 3, 6, and 12 μmol L^−1^ produced an increase of the specific growth rate of *D. quadricauda* of 13, 16 and 7%, respectively; which started to decrease after the exposure to 23 μmol L^−1^ (Figure 1, Table 3).

### Concentration of elements on the nutrient media

We measured the concentrations of Ca and Mn in each medium using ICP-MS. Replete medium contained 48.6 μmol L^−1^ Ca and 7.2 μmol L^−1^ Mn, respectively. Our depletion techniques greatly reduced their concentrations to 3.7 and 0.067 μmol L^−1^ for Ca and Mn, corresponding to 8 and a 1% of the original medium concentration, respectively (see Table 4). In the case of treatments with lanthanides, we tested each medium by exposure to 9.9 or 1.9 μmol L^−1^ of neodymium, as an example of Ln, for Ca and Mn, respectively. There was a serious reduction in the concentration of Nd in each medium after 3 days of algal growth (Table 4).

**Table 4:** **ICP-MS measurements of calcium and manganese in deficient media at 0 and 72 h in the absence or presence of neodymium**.

**minus Nd**				**plus Nd**		
**Time (h)**	**Mn**	**Ca**	**Nd**	**Mn**	**Ca**	**Nd**
**Ca-DEFICIENT MEDIUM**						
0	7.2	3.7	<0.00	7.2	3.7	9.90
72	0.7	6.2	<0.00	1.8	2.4	0.09
**Mn-DEFICIENT MEDIUM**						
0	0.067	48.6	<0.00	0.067	48.6	1.994
72	0.018	43.6	<0.00	0.027	41.1	0.015

All values in μmol L^−1^.

### Growth kinetics

**Figure 2:**
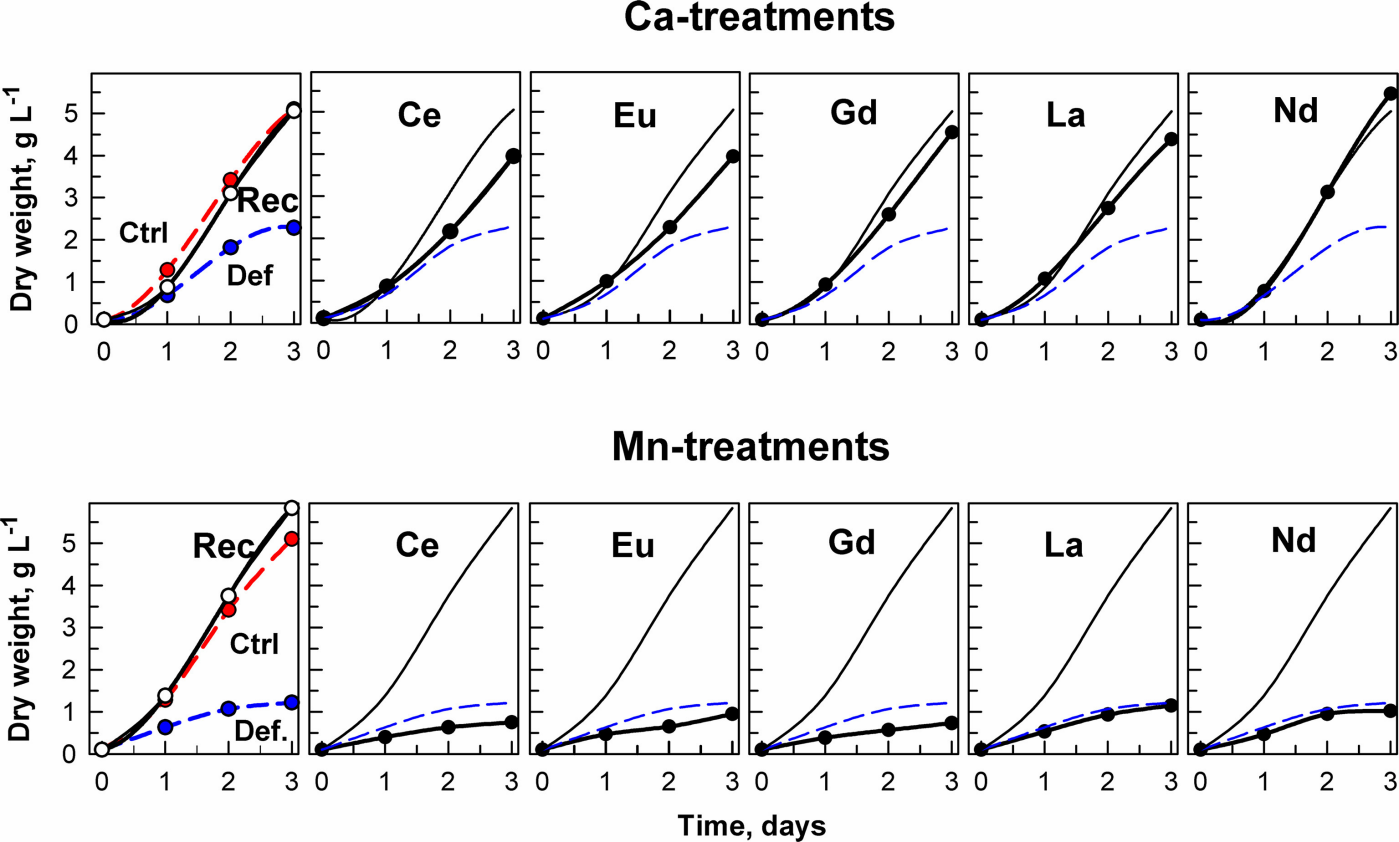
**Changes in dry weight in cultures of the alga *Desmodesmus quadricauda* grown either in complete mineral medium (Ctrl, red symbols, dashed curve) or in calcium- (upper raw of panels) or manganese-deficient mineral medium (bottom raw of panels) (Def., blue symbols, dashed curves)**. To calcium and manganese deficient cultures either the complete mineral medium was added (Rec, black symbols, solid line) or different lanthanides (Ce, Eu, Gd, La, Nd) as marked in individual panels. The curves (without symbols) from recovered (Rec) and deficient (Def) cultures are inserted in panels illustrating the growth in the presence of lanthanides. Supplementary information see Figure S1.

The growth of *D. quadricauda* under complete mineral medium as a control (Ctrl), or under calcium- or manganese-deficient mineral medium is shown in Figure 2. The control and deprived conditions are graphically represented with the red and blue lines, respectively. Our results showed that a deficiency in either metal (independently), but especially Mn^2+^, significantly decreased cellular growth of the microalga (*p* < 0.05) (as “Def” in Figure 2). Re-establishment of the standard medium resulted in recovery of growth in the metal-deprived strain (as “Rec” in Figure 2).

The effect of metal ion-deprivation (Ca^2+^ and Mn^2+^) on the growth of *D. quadricauda* exposed to different lanthanides (at low concentrations) is also shown. In the case of the Ca^2+^-deficient experiment, all Ln treatments increased cellular growth in comparison with the ion-deprived nutrient medium, to reach levels close to the standard conditions (Figure 2). By contrast, none of the Ln treatments alleviated the deleterious effects of Mn deficiency on cellular grow. Instead, the addition of Ce, Eu, and Gd led to a further decrease in cellular growth in the Mn deficient cultures (Figure 2). Significant differences in growth (expressed as dry weight) for the treatments, compared to controls, are shown in Supplementary information Figure S1, Table S1.

### Photosynthetic activity

**Figure 3:**
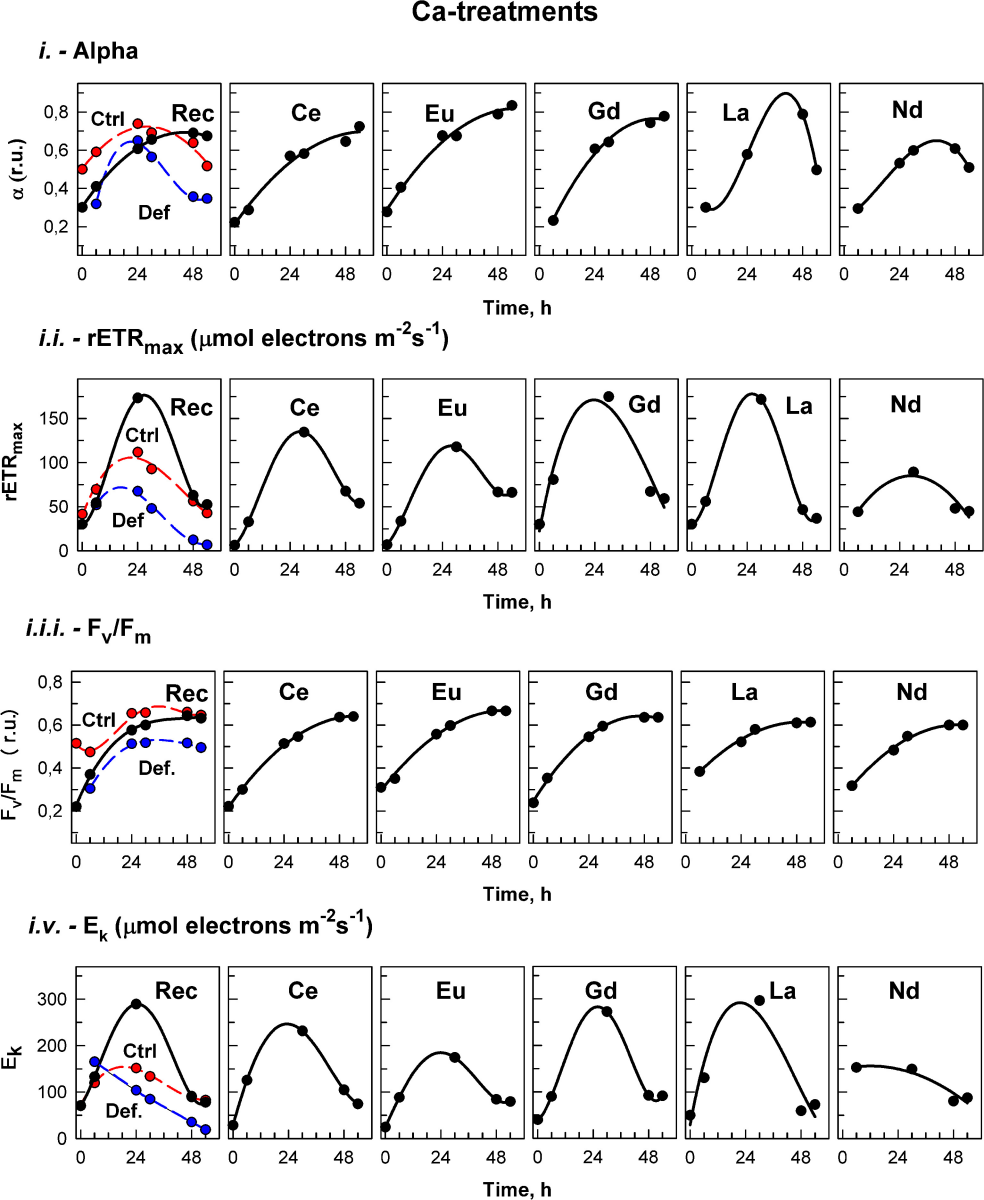
**Calcium treatment**. The photosynthetic parameters in cultures of the alga *Desmodesmus quadricauda* grown either in complete mineral medium (Ctrl, red symbols, dashed curve) or in calcium- deficient mineral medium (Def, blue symbols, dashed curves) are shown. To calcium deficient cultures either the complete mineral medium was added (Rec, black symbols, solid line) or different lanthanides (Ce, Eu, Gd, La, Nd) as marked in individual panels. The photosynthetic parameters were: light-limited photosynthetic efficiency (α); maximum relative electron transport rates (rETR_max_, μmol electrons m^−2^ s^−1^); maximal quantum yield (*F*_*v*_/*F*_*m*_); and light saturation irradiance (E_k_, μmol electrons m^−2^ s^−1^). Supplementary information see Figure S3.

**Figure 4:**
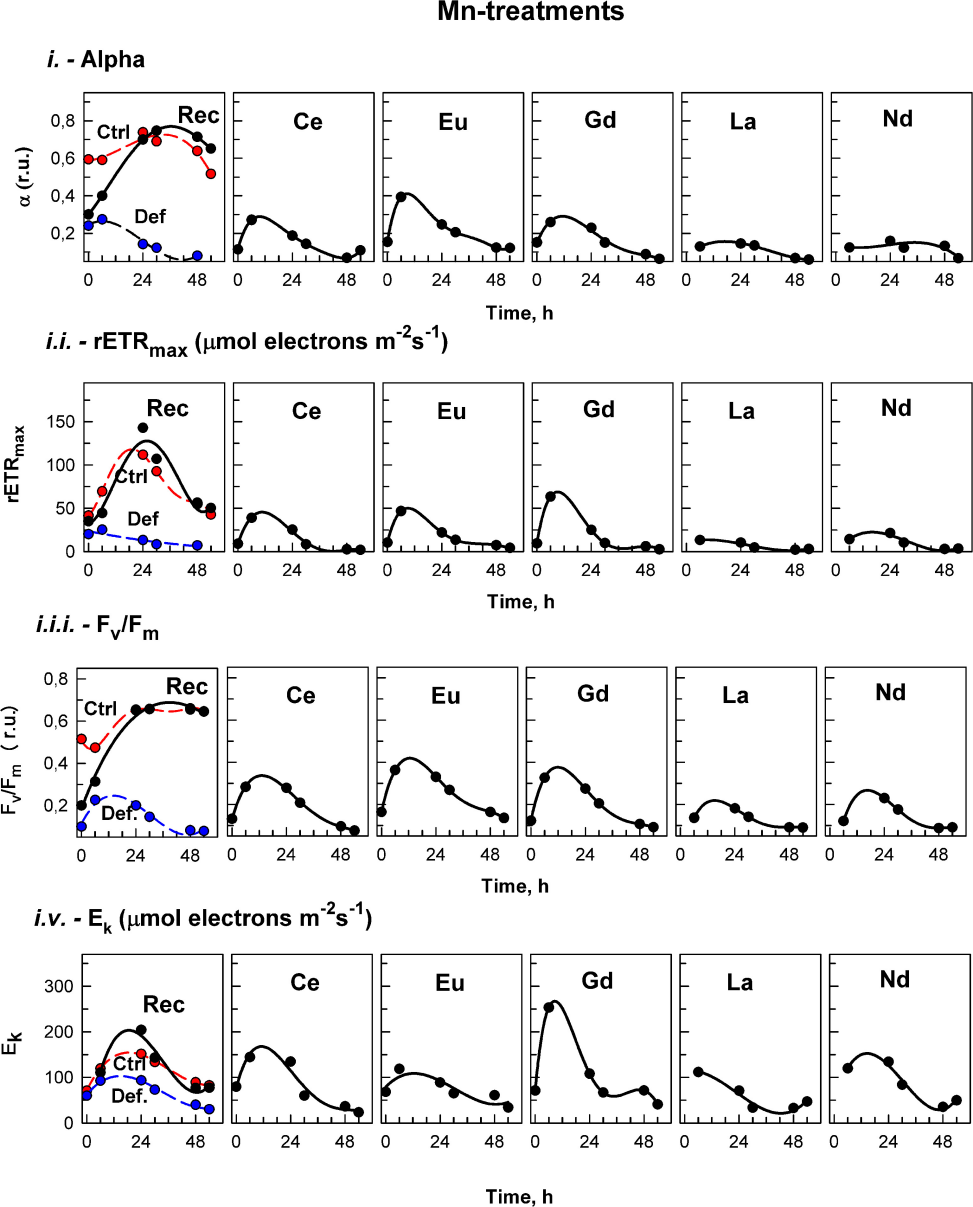
**Manganese treatment**. The photosynthetic parameters in cultures of the alga *Desmodesmus quadricauda* grown either in complete mineral medium (Ctrl, red symbols, dashed curve) or in manganese- deficient mineral medium (Def blue symbols, dashed curves) are shown. To manganese deficient cultures either the complete mineral medium was added (Rec, black symbols, solid line) or different lanthanides (Ce, Eu, Gd, La, Nd) as marked in individual panels. The photosynthetic parameters were: light-limited photosynthetic efficiency (α); maximum relative electron transport rates (rETR_max_, μmol electrons m^−2^ s^−1^); maximal quantum yield (*F*_*v*_/*F*_*m*_); and light saturation irradiance (E_*k*_, μmol electrons m^−2^ s^−1^). Supplementary information see Figure S3.

The effects of treatment on *in vivo* chlorophyll fluorescence of *D. quadricauda* are shown in Figures 3, 4, and Table 5. Under complete mineral medium, the maximum quantum yield of PSII (*F*_*v*_/*F*_*m*_) showed no significant differences (*p* < 0.05) between the first and second days. Under these standard (replete) conditions, the *F*_*v*_/*F*_*m*_ mean value was 0.66 ± 0.00 (Table 5). However, omission of either Ca^2+^ or Mn^2+^ from the culture medium significantly decreased the maximum quantum yield. These nutrient stresses (metal-limited conditions) in microalgae were detected by a 21% ± 0.05 decrease (Ca^2+^) or 88% ± 2.50 decrease (Mn^2+^) compared with the controls (mean ± SD, *n* = 3; Figures 3, 4, respectively). In the first 6 h, stresses produced by sampling, centrifugation and nutrient medium replacement was observable in all tests (control and treatments), with recovery within the first 24 h. Only in the case of Ca^2+^ (Figure 3), but not Mn^2+^-deficiency (Figure 4), did the addition of low concentrations of Ln^3+^ produce a recovery in *F*_*v*_/*F*_*m*_ and apparently alleviated the symptoms of an ion-deficit.

**Table 5:** **Summary of the physiological parameters of *Desmodesmus quadricauda* measured under replete conditions (standard medium as control, “Ctrl”) and under selective nutrient stress (deficient condition, “Def”: Ca^2+^-deficient media “a” and Mn^2+^-deficient media “b”)**.

**Parameters**	**Standard conditions**							
	**Ctrl**	**Rec**	**Def**	**La^3+^**	**Ce^3+^**	**Nd^3+^**	**Eu^3+^**	**Gd^3+^**
**(a) CALCIUM-DEFICIENT MEDIA**								
*F*_*v*_/*F*_*m*_	0.66	0.64	0.52	0.66	0.64	0.64	0.67	0.66
rETR_max_	56.25	62.77	12.30	46.62	67.60	47.93	66.48	67.02
E_k_(μmol m^−2^ s^−1^)	89.29	91.09	35.30	59.71	104.82	80.18	84.41	91.92
α	0.64	0.69	0.36	0.79	0.65	0.61	0.79	0.74
**(b) MANGANESE-DEFICIENT MEDIA**								
*F*_*v*_/*F*_*m*_	0.66	0.65	0.08	0.09	0.18	0.09	0.19	0.17
rETR_max_	56.25	54.65	0.00	2.11	2.49	2.78	7.36	5.99
E_k_(μmol m^−2^ s^−1^)	89.29	76.57	0.00	32.76	36.57	35.19	60.96	71.33
α	0.64	0.71	0.00	0.07	0.07	0.13	0.12	0.09

The effect of the re-establishment of standard medium (recovery, “Rec”) or by adding a low concentration of different Ln is described. Maximal quantum yield (Fv/Fm) and the ETR parameters obtained from the rapid light curves as maximal relative ETR (rETR_max_), photosynthetic efficiency (α) and saturated irradiance (E_k_) at 48 h are shown. Values are means (n = 3), with a complete listing, SD and statistics in Supplementary information Figure S3, Table S2.

rETR_max_ values, determined from the RLCs, were significantly higher (*p* < 0.05) in the first hours under standard than under Ca^2+^-deficient conditions (“Def” see Supplementary information Figure S3). After 30 h, the rETR_max_ increased in all Ln treatments under Ca^2+^-deficiency, reaching values significantly higher (*p* < 0.05) than in the controls (Figure 3). After that, values started to decrease, and although after 48 h, they were significantly higher than values under deficiency conditions (Def), no significant differences were found compared to the control.

On the other hand, treatments of the algae with Ln, under Mn^2+^-deficiency, showed lower rETR_max_ values compared to those of the deficiency conditions during the entire experiment (Figure 4, Table 5).

Photosynthetic efficiency (α), obtained from the RLCs, was significantly higher in cultures grown in standard nutrient medium than under either ion-deficiency conditions, or in Mn^2+^-deficient + Ln^3+^ treatments. Almost no differences were observed between the Mn^2+^-deficient control and those after exposures to Ln^3+^ (Figure 4). However, under Ca^2+^-deficient treatments exposed to Ln^3+^, significant differences were observed. While in both controls, the photosynthetic efficiency (α) tended to decrease, cultures in Ca^2+^-deficient treatments exposed to Ce, Eu, and Gd achieved photosynthetic efficiency values significantly higher than those observed in the controls (Figure 3).

The saturated irradiance (E_k_), obtained from the RLCs, showed significant differences between standard conditions and both deficiency treatments (Supplementary information Figure S3). E_k_ tended to increase within the first 24–30 h and after that a decrease was observed in all treatments under both nutrient deficiency conditions. In general, no significant differences were found between Ca^2+^-deficiency treatments and controls, although at certain times, when maximum values were achieved in some treatments, values were higher than those in the controls (see Figure 3). For Mn^2+^-deficiency treatments, values were similar to those in the controls in the first hours although in some of the treatments i.e., Ce, Eu, La and Nd, they were lower than those in the control at the end of the experiment (Figure 4).

Replacement of the deficient medium (either Ca^2+^ or Mn^2+^) with standard conditions produced a significant recovery, observable in all fluorescence (*F*_*v*_/*F*_*m*_, rETR_max_, α, and E_k_) parameters (Figures 3, 4).

## Discussion

In preliminary experiments, we confirmed that at lower concentrations (<25 μmol L^−1^), Ln can produce stimulatory effects on growth of *D. quadricauda* (Figure 1, Table 3). Within this range we were able to establish metal-deficiency experiments. *ŠS-medium* is considered as a rich mineral medium (Zachleder and Šetlik, 1982), where cells may be able to accumulate metals. We initiated this experiment from an agar-plate culture, transferred it (±0.0005 g) into deprived-liquid medium and maintained it as a pre-culture for 2 weeks to effectively reduce the cellular content of the target metals (Table 4). Metal deficiency can, in this way, be induced at levels sufficient to partially inhibit physiological processes (e.g., to reduce photosynthesis), but not severe enough to reduce the survival of the population (Figure 2). The goal was not to grow algae in the complete absence of Ca^2+^ or Mn^2+^, which has previously been studied (see Dvořáková-Hladká, 1976; Adam and Issa, 2000), but to observe if Ln may compensate for levels of deprivation in microalgae. Under Ca^2+^-deficient conditions, certain Ln were able to partly alleviate the symptoms of the deficiency: Increased growth and biomass production, to almost normal physiological levels for microalgae observed under standard conditions (Table 5). This was not true for any of those metals under Mn^2+^-deficient treatments, where the deleterious effect on cellular growth and photosynthetic competence was increased even more (Figure 2).

Using ICP-MS, we have analyzed the content of Ca and Mn both in deficient mineral media, and in Ca- and Mn-deficient culture media after exposure to one lanthanide, Nd. The contents of both elements (Ca, Mn) in the deficient media were extremely low (Ca 3.7 and Mn 0.07 μmol L^−1^), and would not be sufficient to support algal growth from 100 mg DW L^−1^ to more than 5000 mg DW L^−1^, which were the values achieved in control and recovery cultures (curves Ctrl and Rec, Figure 2). Consequently, growth was limited substantially by both deficiency-treatments. Cells grew slowly for about 2 days and then stopped growth completely (compare curves Ctrl and Def in Figure 2).

After adding complete mineral medium (for both cases of deprivation) or by exposure to REEs (only in the case of Ca-deficiency) growth recovered was substantial and in some cases near the level of the control culture (Figure 2). The amount of Nd measured in the medium after 3 days of algal growth decreased from 9.9 μmol L^−1^ to 0.09 μmol L^−1^ (Table 4). Although we don't know the exact bioavailable concentration for each lanthanide, if we consider the growth response to lanthanides at low levels of Ca, and if we use as a reference the values of the ICP-MS measurement of Nd, we strongly suggest that REEs can substitute for some functions of the missing (Ca) ions.

### Metal replacement

Ln are non-essential elements that have been shown to produce diverse physiological effects (Jin et al., 2009; Tai et al., 2010). In the last two decades, they have been suggested to play possible roles in terrestrial organisms as replacements for essential elements like Ca^2+^, Mg^2+^, and Mn^2+^ (Brown et al., 1990; Squier et al., 1990). This was also observed for microalgae in our Ca^2+^ treatment experiments (Figure 3), but further studies are needed to identify specific mechanisms of action. In this sense, Wang et al. (2003) stated that the biological behavior of non-essential metal ions like Ln^3+^ originates from the principle of analogy to an essential metal ion. Their properties are *close to* but *not the same* as the original. Therefore, displacement (by Ln) can have different consequences depending on the role of the native metal, possibly explaining why, in some cases, functionality can be retained (Yocum, 2008). This could explain our present (and divergent) results when exposing Ca^2+^-deficient and Mn^2+^-deficient algae to lanthanides (Figures 2–4).

### Essential elements

Of all the metals, Ca^2+^ may exert the widest range of effects on biological systems, including functions related to structure, regulation of enzymatic activity and intercellular and intracellular signaling (Brand and Becker, 1984; Yocum, 2008). In previous studies with microalgae under Ca^2+^ deficient conditions, photosynthetic oxygen evolution and respiratory oxygen uptake were severely affected, in parallel with growth rate and chlorophyll content (Adam and Issa, 2000). These authors suggested that many enzymes (e.g., arginine kinase, adenosine triphosphatase, adenylate kinase) that are regulated by this metal are directly involved in vital processes such as photosynthesis and respiration. Dvořáková-Hladká (1976) also associated Ca^2+^ with energy, nitrogen and phosphorus metabolism of microalgae such as *Scenedesmus obliquus*. This could explain the observed decrease in physiological state of the deprived green algae (Figures 2–4).

Manganese (and Ca^2+^) exist in the oxygen-evolving complex of plant and algal PSII, and participate in the water-splitting reaction; moreover, they could be involved in maintenance of the chloroplast structure. Photosynthetic water oxidation takes place at a catalytic Mn(4)-Ca site within the oxygen-evolving complex of PSII, which is embedded in the thylakoid membranes of green plants, cyanobacteria and algae (Yachandra and Yano, 2011; Hou and Hou, 2013). Mn^2+^ can be also a redox cofactor or activator at metal-binding sites of many enzymes and coenzymes (e.g., manganese superoxide dismutase), so the redox balance and PSII are expected to be the prime targets of Mn^2+^-deficiency in photosynthetic organisms (Cao et al., 2011). The important role of these two metals in algae, and the effects of deficiencies on algal physiology (e.g., slow growth, structural alterations, accumulation of less chlorophyll, loss of PSII and enzyme activity), have been previously reported for a few marine phytoplanktonic species and freshwater green microalgae (Constantopoulos, 1970; Dvořáková-Hladká, 1976; Adam and Issa, 2000; Allen et al., 2007; Cao et al., 2011; Hsieh et al., 2013, and references therein).

### Physiological responses

Physiological stress by nutrient limitation was specifically measured not only by declines in cellular growth and reproduction, but also in terms of photosynthetic parameters. PAM fluorometry has been shown to be an effective method to study stress (see Komenda, 1998; Mallick and Mohn, 2003; White et al., 2011; Figueroa et al., 2013; Giovanardi et al., 2014), and in this work, demonstrated a significant decrease in *F*_*v*_/*F*_*m*_, rETR_max_, E_k_, and α in comparison with standard conditions (Table 5, Figures 3, 4). For comparison, values of *F*_*v*_/*F*_*m*_ expressed for different species of the genus *Desmodesmus* under different standard nutrient media ranged from 0.60 to 0.74 (see Komenda, 1998; Koblížek et al., 2001; Karsten et al., 2007; Hu et al., 2013; Samorí et al., 2013). Interestingly, by adding individual Ln to the Ca^2+^-deficient treatment we observed different, although irregular, increases in *F*_*v*_/*F*_*m*_, rETR_max_, E_k_, and α, toward the reestablishment of standard conditions in the control (Ctrl). This is surprising because previously, it was demonstrated that although Ln are successful competitors with Ca^2+^ for binding sites in PSII, none of them retained functionality and treatment did not result in reactivation of oxygen evolution activity (Ghanotakis et al., 1985; Bakou et al., 1992; Bakou and Ghanotakis, 1993; Ono, 2000; Yachandra and Yano, 2011). There is some support for our observed Ln^3+^ stimulation of the growth of the control cultures (Figure 1) and Ca-limited treatments (Figure 2). Kruk et al. (2003) described a possible stimulation of oxygen evolution in PSII membranes by low concentrations of Eu^3+^ and Dy^3+^ ions. Under other nutrient-deficient conditions, Huang et al. (2008a,b) and Liu et al. (2008) demonstrated alleviation effects of Ce^3+^ on the photosynthetic rate (electron transport rate) of spinach chloroplasts under Ca^2+^-deficiency and the same metal improved ETR and yield values of Mg^2+^-deficient maize (Zhao et al., 2012). The stimulatory effect (or reduced stress) on *D. quadricauda* could be derived from secondary processes affecting algal physiology, but, because Ca^2+^ has a multifaceted role in photosynthesis (see Brand and Becker, 1984), it will be necessary to undertake a detailed molecular study. It has been suggested that Ln could modulate plant photosynthesis by interactions with K^+^, Na^+^, or Ca^2+^, ribulose-1,5-bisphosphate carboxylase/oxygenase, oxidative damage and redox systems, and indolyl-acetic acid (Chen et al., 2000; Wang et al., 2011, and references therein).

In the case of the Mn^2+^-deficiency treatments of *D. quadricauda*, there was no such stimulation by adding Ln to the deficient medium. Furthermore, in many cases the algae were more stressed than under deficient conditions, as was reflected by even lower values for the photosynthetic parameters (Figure T4). There is only one previous report of Mn^2+^-deficient maize treated with Ce^3+^ (Qu et al., 2012). The authors demonstrated that the Ln could significantly relieve reductions in *F*_*v*_/*F*_*m*_, Y(I and II), ETR(I and II) and the photochemical quenching coefficient (*q*P), among other parameters, as compared to those of the control. They suggested that Ce^3+^ may improve the function of PSI and PSII under Mn^2+^-deprived stress, although the mechanisms are unknown. In our work, we did not observe any improvement. Furthermore, although certain chemical similarities with Ln, this metal serves a key redox role in water oxidation in the Mn4 clusters of PSII (Hou and Hou, 2013), a highly specific function that other metals are probably not able to duplicate.

### Environmental consequences

We want to make clear that these are simulation experiments where most of the conditions are controlled. We used a monoculture, a standard enriched medium, bubbling with a high CO_2_ level, and concentrations of Ln that were picked subjectively (related to algal behavior and the original concentration of the omitted metal). In this way, although it gave us valuable hints to study the effects of Ln on algae, it is by no means an ecological or environmental study, requiring more natural conditions.

Lanthanides represent a potential environmental threat, particularly in high metal-exposure areas such as sites for mining, refining and recycling of REEs (Tyler, 2004). As these elements have become indispensable for a number of critical technologies, the demand for REEs in the next few years is expected to increase (Loell et al., 2011). Similarly to trace elements, Ln exhibit both positive and negative effects on algal growth and development, at low concentrations and high concentrations, respectively (Chen et al., 2000). Nevertheless, as we have demonstrated, their behavior is not simple, and in certain cases, even at low concentrations, Ln can be toxic to microorganisms. These and further studies are essential to understand the physiological and ecological effects that Ln produce in nature.

## Conclusions

At low concentrations, lanthanides Ln can produce a stimulatory effect on the growth of microalgae. These non-essential elements may replace certain metals in a few physiological roles, as was demonstrated by alleviation of Ca^2+^-deficiency in our experiments. It is not yet clear which pathways were affected by the metals, and at what stage they became either stimulatory or toxic. The same organism responds differently to the same non-essential element, depending on the cellular physiological state. This means that, depending on the stress that algae were suffering at any specific time, the same Ln concentration could have stimulatory effects, or may increase deleterious effects and finally suppress growth; this calls into question the safety of Ln at low concentrations.

## Author's statement

The work has not been published previously (except in the form of an abstract), and all authors have approved the final article.

## Author contributions

Conceived and designed the experiments: Celia G. Jerez, Félix L. Figueroa, Franz Goecke, Kateřina Bišová, Tomáš Řezanka, Milada Vítová, Vilém Zachleder. Performed the experiments: Franz Goecke. Analyzed the data: Franz Goecke, Celia G. Jerez, Félix L. Figueroa, Milada Vítová, Tomáš Řezanka. Contributed reagents/materials/analysis tools: Félix L. Figueroa, Kateřina Bišová, Milada Vítová, Tomáš Řezanka, Vilém Zachleder. Wrote the article: Franz Goecke, Celia G. Jerez, Félix L. Figueroa, Kateřina Bišová, Milada Vítová, Vilém Zachleder. Graphics: Franz Goecke, Celia G. Jerez, Kateřina Bišová, Vilém Zachleder. Final approval of the version to be submitted; Celia G. Jerez, Félix L. Figueroa, Franz Goecke, Kateřina Bišová, Milada Vítová, Tomáš Řezanka, Vilém Zachleder.

### Conflict of interest statement

The authors declare that the research was conducted in the absence of any commercial or financial relationships that could be construed as a potential conflict of interest.

## Supplementary material

Table S1 **Raw data of growth kinetics and statistics**.

Table S2 **Raw data of the photosynthetic parameters and graphics**.

## Abbreviations

α: photon-capturing efficiency of PSII in the light limited range
dw: dry weight
E_k_: light saturation irradiance
ETR: electron transport rate
*F*_*v*_/*F*_*m*_: maximal quantum yield of PSII photochemistry
Ln: lanthanides
PAM: pulse amplitude modulated fluorometry
PSII: photosystem II
REE: rare earth element.
