# Supplementary material for: Use of lanthanides to alleviate the effects of metal ion-deficiency in Desmodesmus quadricauda (Sphaeropleales, Chlorophyta)
Source: Front Microbiol. 2015 Jan 28;6:2. doi: 10.3389/fmicb.2015.00002 (PMC4309186; doi:10.3389/fmicb.2015.00002)
Supplement: Supplementary file 3 [file Image1.PDF]

Supplementary Materials

FIGURE S1 (from Figure 2 in text)

Growth bars for the microalga *Desmodesmus quadricauda* obtained for 3 days under standard conditions, under metal-deprivation media (Def) and also after exposure to selected lanthanides (values in mg mL^{-1} dry weight and cenobia x 10^5 mL^{-1}, respectively). Metal-deprivation consisted of deficiency of calcium (a-b) or manganese (c-d). We also replaced the deficient medium with standard medium to determine whether there was a recovery to standard conditions (Rec). Significant differences from the deficient conditions (Def) and the control, of dry weight are displayed by (+) and (*), respectively. For each sample, values are displayed as means ± SD (n>3).

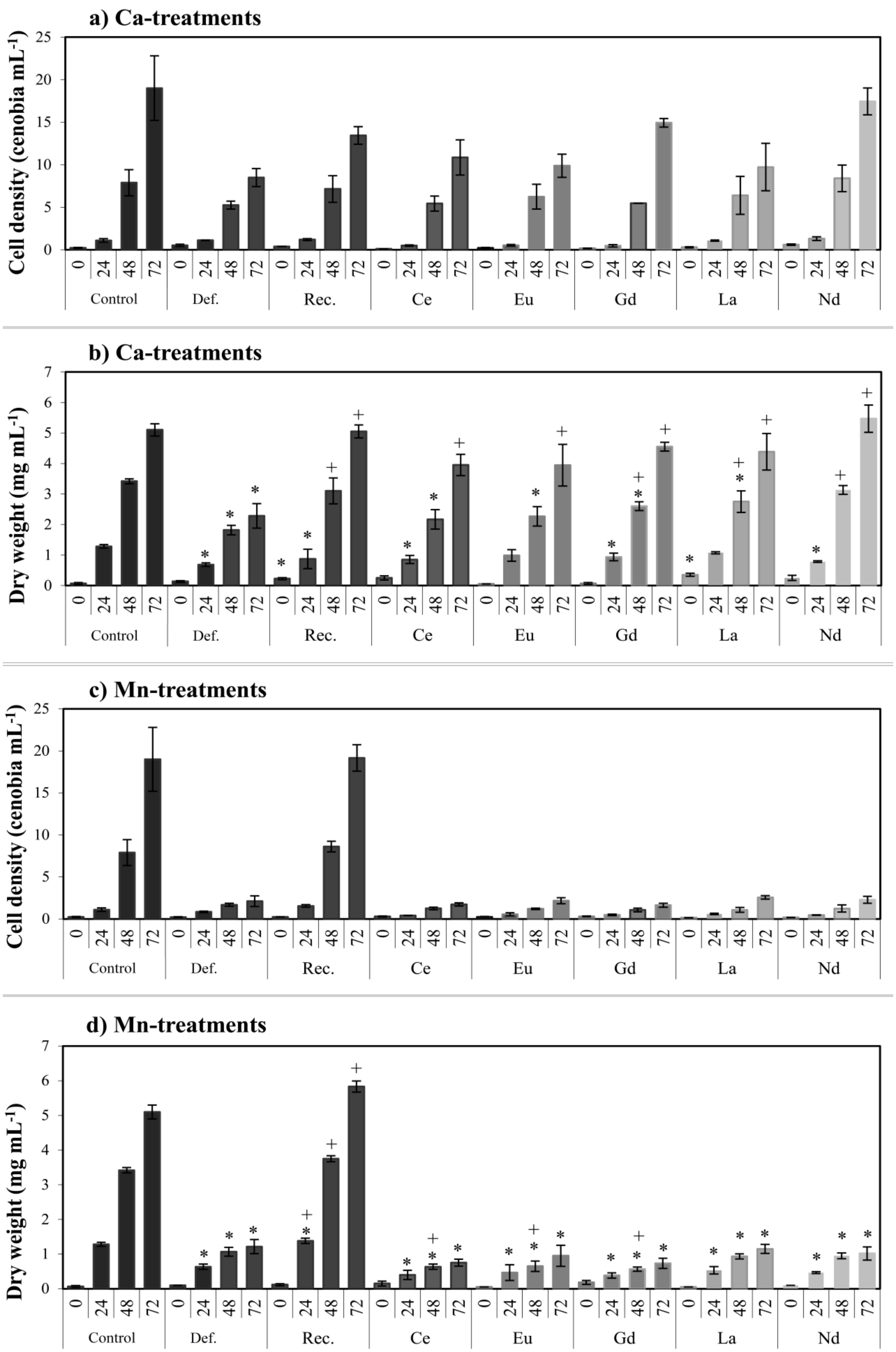

a) Ca-treatments
Cell density (cenobia mL-1)
b) Ca-treatments
Dry weight (mg mL-1)
c) Mn-treatments
Cell density (cenobia mL-1)
d) Mn-treatments
Dry weight (mg mL-1)
0
24
48
72
Control
Def.
Rec.
Ce
Eu
Gd
La
Nd
