# Supplementary material for: Use of lanthanides to alleviate the effects of metal ion-deficiency in Desmodesmus quadricauda (Sphaeropleales, Chlorophyta)
Source: Front Microbiol. 2015 Jan 28;6:2. doi: 10.3389/fmicb.2015.00002 (PMC4309186; doi:10.3389/fmicb.2015.00002)
Supplement: Supplementary file 4 [file Image2.PDF]

Supplementary Materials

FIGURE S3 (from Figure 3 in text)

Treatment of Calcium deficiency (**a**) and treatment of Manganese deficiency (**b**). The photosynthetic parameters of each treatment and their significant differences from the deficient conditions and controls are displayed by (+) and (*), respectively. Light-limited photosynthetic efficiency (α), maximum relative electron transport rates ($rETR_{max}$, μmol electrons m^{-2} s^{-1}), maximal quantum yield (F_v/F_m), and light saturation irradiance (E_k, μmol electrons m^{-2} s^{-1}), as means ± SD, and as a function of nutrient availability: standard medium, Ca^{2+} or Mn^{2+} deficiency (Def.) and reestablishment of standard conditions or “recovery” (Rec), and the presence of different lanthanides.

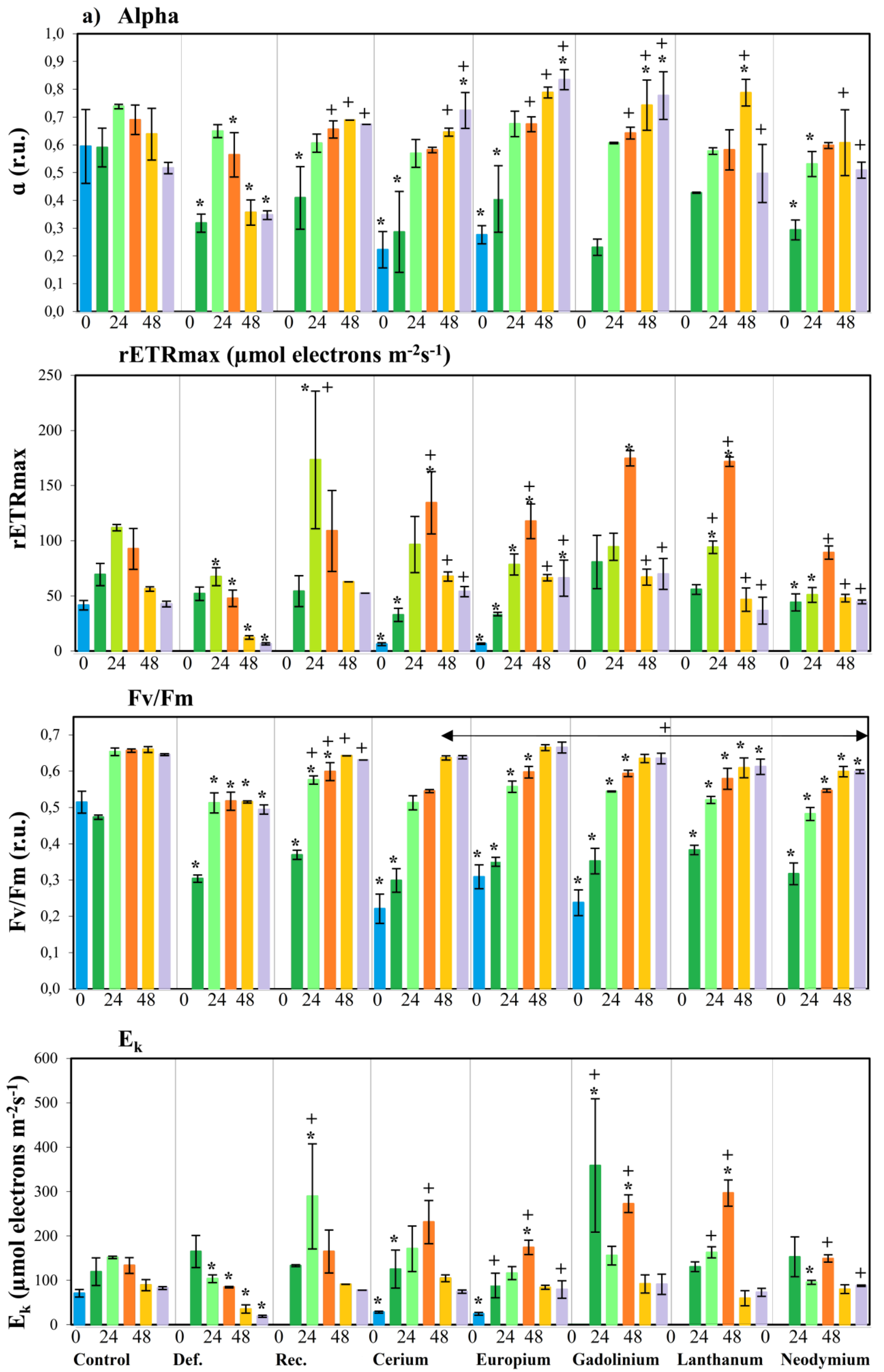

a) Alpha
α (r.u.)
rETRmax (µmol electrons m-2s-1)
rETRmax
Fv/Fm
Fv/Fm (r.u.)
Ek
Ek (µmol electrons m-2s-1)
0 24 48
Control
Def.
Rec.
Cerium
Europium
Gadolinium
Lanthanum
Neodymium

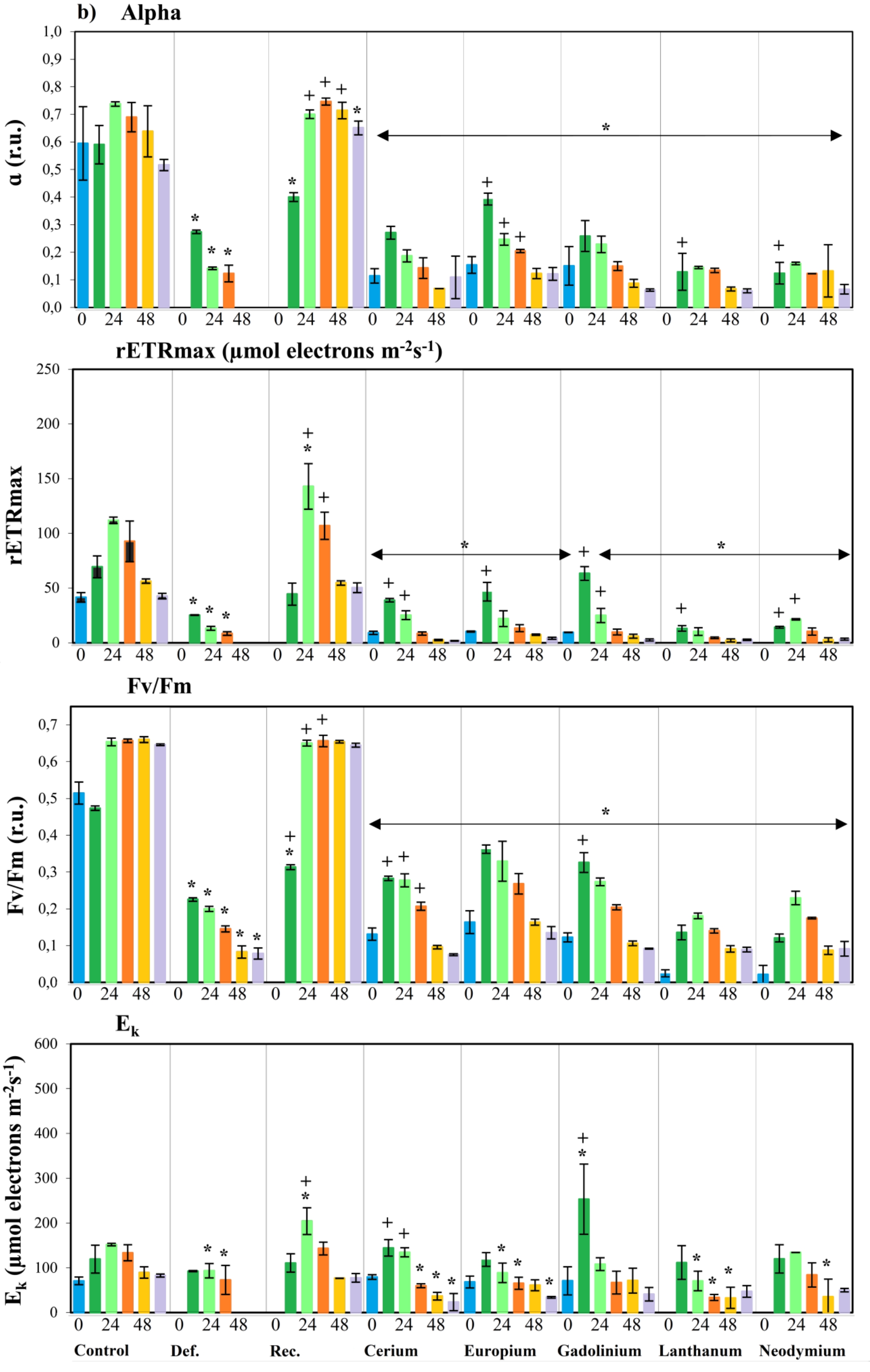
b) Alpha
α (r.u.)
rETRmax (µmol electrons m-2s-1)
rETRmax
Fv/Fm
Fv/Fm (r.u.)
Ek
Ek (µmol electrons m-2s-1)
0 24 48
Control
Def.
Rec.
Cerium
Europium
Gadolinium
Lanthanum
Neodymium
